# Emerging Therapeutic Approaches for Modulating the Intestinal Microbiota

**DOI:** 10.3390/pharmaceutics18020197

**Published:** 2026-02-03

**Authors:** Ilaria Cosimato, Annalisa Brescia, Gianluigi Franci, Vincenzo Casolaro, Veronica Folliero

**Affiliations:** 1 Department of Medicine, Surgery and Dentistry “Scuola Medica Salernitana”, University of Salerno, 84081 Baronissi, Italy; ilariacosimato06@gmail.com (I.C.); gfranci@unisa.it (G.F.); vcasolaro@unisa.it (V.C.); 2 Immunodiagnostics Unit, San Giovanni di Dio e Ruggi d’Aragona University Hospital, 84126 Salerno, Italy; annalisa.brescia@gmail.com; 3 Microbiology and Virology Unit, San Giovanni di Dio e Ruggi d’Aragona University Hospital, 84126 Salerno, Italy

**Keywords:** microbiota, dysbiosis, therapeutics, FMT, BCT, OMVs

## Abstract

**Background/Objectives**: The gut microbiota is increasingly recognized as a key determinant of human health, playing a vital role in metabolism, immunity, and disease susceptibility. Dysbiosis, or microbial imbalance, is associated with gastrointestinal disorders such as irritable bowel syndrome (IBS), inflammatory bowel disease (IBD), and *Clostridioides difficile* infection (CDI), as well as extraintestinal conditions, including obesity, cardiovascular disease, and neuropsychiatric disorders. This review aims to provide an updated overview of emerging therapeutic strategies to modulate the gut microbiota to restore eubiosis and improve health outcomes. **Methods**: A narrative review of recent literature was conducted, focusing on preclinical and clinical studies investigating microbiota-targeted therapies. The review primarily covers innovative interventional approaches, including fecal microbiota transplantation (FMT), bacterial consortium transplantation (BCT), bacteriophage therapy and outer membrane vesicles (OMVs). **Results**: Evidence supports the role of probiotics, prebiotics, and synbiotics in remodeling microbial communities and improving host health, although their effects may be strain- and context-dependent. FMT has demonstrated high efficacy in the treatment of recurrent *Clostridium difficile* infections and is being studied for IBD, IBS and extraintestinal diseases, following the recent Food and Drug Administration approval of the first commercial FMT products. BCT offers a standardized alternative to donor-derived material, with early clinical successes such as FDA-approved SER-109. Phage therapy and OMVs represent promising frontiers, offering targeted microbial modulation and interactions with the immune system, although clinical data remain limited. **Conclusions**: Emerging gut microbiota modulation strategies offer new perspectives for precision medicine and could transform the prevention and treatment of many diseases, but further studies are needed to ensure their safety, standardization, and clinical application.

## 1. Introduction

The concept of microbiota first appeared in the early 20th century, coined to describe the communities of microorganisms that colonize the human body from the skin and respiratory tract to the oral cavity, and particularly the gut [1]. Among these niches, the gut microbiota is considered the most significant for human health due to its remarkable density, taxonomic diversity, and the wide range of biological functions it performs in close interaction with the host [2]. Over the past two decades, gut microbiota has emerged as one of the most dynamic and rapidly expanding topics of biomedical research, recognized as a central participant in human physiology and in the pathogenesis of a wide spectrum of diseases [3]. The human gut hosts trillions of bacteria, viruses, fungi, and archaea, constituting a dynamic and complex ecosystem that has co-evolved with humans, establishing a finely balanced mutualistic relationship [4]. Human guts host a complex and dynamic ecosystem composed of bacteria, viruses, fungi, and archaea, coevolving with humans in a finely tuned mutualistic relationship. Interactions between these microorganisms shape community composition, metabolic functions, and host health. Bacteria and fungi establish both competitive and cooperative relationships, sharing metabolites and contributing to microbial diversity. Bacteriophages impact bacterial composition through selective infection, with cascading effects on microbial metabolism. Collectively, these interactions can influence the integrity of the intestinal barrier, the immune response, and systemic metabolism, determining whether the host-microbiota balance is maintained or evolves toward dysbiosis. Generally, gut microbiota is composed of six phyla, including *Firmicutes*, *Bacteroidetes*, *Actinobacteria*, *Proteobacteria*, *Fusobacteria*, and *Verrucomicrobia*. Among the most represented fungi are *Candida*, *Saccharomyces*, *Malassezia*, and *Cladosporium*. Viruses include *Astroviruses* and *Caliciviruses*, phages include *Myoviridae*, *Podoviridae*, and *Siphoviridae*, archaea are primarily *Methanobrevibacter smithii* and protozoa include *Entamoeba* and *Blastocystis* [5,6]. The composition of the gut microbiota is heterogeneous across the gastrointestinal tract and is influenced by intrinsic factors, such as host genetics, as well as environmental influences, including diet, exposure to xenobiotic substances, infections, and drug treatments [7]. Under physiological conditions, this ecosystem serves essential functions: fermentation of otherwise indigestible substrates with production of short-chain fatty acids (SCFAs), biosynthesis of vitamins, regulation of micronutrient absorption (Ca, Mg, Fe), development and maintenance of the intestinal epithelium, enhancement of the mucosal barrier, protection against pathogens, and modulation of immune responses [8]. Increasing evidence shows that the microbiota interacts closely with innate epithelial, myeloid, and lymphoid cells, generating feedback mechanisms that affect both the composition of the microbial community and the physiology of the host [9]. In healthy individuals, the host and its microbiota coexist in a mutualistic balance; however, this balance can be disrupted by environmental or clinical factors, leading to dysbiosis [10]. Dysbiosis is typically characterized by decreased microbial diversity and an imbalance between beneficial and opportunistic species [11,12]. It can compromise the integrity of the intestinal barrier, alter bile acid metabolism, reduce production of such SCFAs as butyrate and propionate, and trigger inflammatory signals, thus predisposing individuals to a wide range of pathological conditions [13,14]. The most direct consequences are observed in the gastrointestinal tract, leading to disorders such as irritable bowel syndrome (IBS), inflammatory bowel disease (IBD), and *Clostridioides difficile* infection (CDI) [15]. However, a growing body of evidence implies dysbiosis in extraintestinal pathologies as well, including obesity, metabolic syndrome, type 2 diabetes, cardiovascular disease, autoimmune disorders, and neuropsychiatric conditions. In this context, a connection between the gut microenvironment and mitochondrial function has been demonstrated in the pathophysiology of numerous diseases. Intestinal dysbiosis and increased intestinal permeability impact mitochondrial function through increased LPS and decreased butyrate production, affecting glial and immune cells. This leads to the activation of oxidative stress pathways and ceramide production, along with reduced orexin and melatonin, compromising oxidative phosphorylation and circadian rhythms. The gut-mitochondria axis emerges as a key mechanism in the regulation of systemic homeostasis and various diseases [16]. Together, these findings support the concept of the microbiota as a “virtual organ” that exerts systemic effects on host health [17]. The association of specific microbiota compositions with states of health and disease has been made possible thanks to the development of advanced molecular technologies, such as 16S rRNA gene sequencing, whole-genome shotgun sequencing, and integrated multi-omic approaches [18]. The application of metagenomics, metatranscriptomics, and metabolomics, combined with the use of artificial intelligence (AI) for large-scale data analysis, has revolutionized our ability to characterize the gut ecosystem and its interactions with the host [19]. While early research focused mainly on the role of microbiota in disease pathogenesis, more recent studies on healthy individuals have shown that dietary modifications and targeted interventions, such as probiotics, prebiotics, and synbiotics, can beneficially modulate microbial communities [20]. These interventions aim to maintain or restore eubiosis, defined as a dynamic state of equilibrium in the microbiota characterized by a microbial composition and functionality capable of supporting host homeostasis, intestinal barrier integrity, and an appropriate immune response. In addition to traditional nutritional approaches, innovative approaches such as fecal microbiota transplantation (FMT), synthetic bacterial consortia, and the use of bacteriophages and outer membrane vesicles (OMVs) have attracted considerable interest [21,22,23]. While differing in their mechanisms of effect and levels of standardization, these strategies share the overall goal of re-establishing a balanced gut ecosystem and mitigating the deleterious effects of dysbiosis on host health. This review provides an up-to-date overview of emerging strategies for gut microbiota modulation. We examine their biological rationale, summarize preclinical and clinical evidence, and discuss their potential benefits and limitations. In short, these approaches can represent a major step forward in precision medicine, offering new opportunities to transform the prevention and treatment of a wide range of chronic and infectious diseases through targeted manipulation of the microbial ecosystem.

## 2. Gut Microbiota in Health and Dysbiosis

Human guts harbor a highly diverse microbial community [24]. Numerous studies have demonstrated the crucial role of the gut microbiota in regulating physiological processes, including metabolism, immunity, and barrier function [25]. Under healthy conditions, the gut microbiota maintains stability, resilience, and a symbiotic relationship with the host. Vertebrate hosts and their gut microbes form interconnected, dynamic systems that maintain homeostasis [26]. Disruption of this balance often manifests initially in the gastrointestinal tract. Dysbiosis can, in fact, induce the proliferation of pathogenic microorganisms, which generate toxic metabolites, compromise intestinal barrier integrity, facilitate translocation of harmful substances into the bloodstream, and trigger local or systemic inflammatory responses [27]. In addition, an imbalanced microbiota can modulate immune function by activating or suppressing immune pathways, resulting in chronic inflammation and immune dysregulation. These alterations underline the pathogenesis of various disorders, including IBS and IBD [28].

### 2.1. Intestinal Diseases

Significant alterations in the composition of the intestinal microbiota are found in various pathological conditions of the gastrointestinal tract. Numerous studies indicate that CDI is closely associated with marked intestinal dysbiosis, characterized by a significant reduction in microbial diversity and structural alterations in the bacterial community [29]. In healthy individuals and CDI carriers, the gut microbiota is characterized by an abundance of *Bifidobacteria* and a lower presence of *Proteobacteria*. In contrast, in patients with CDI, dysbiosis manifests with an increase in *Lactobacillaceae* and *Enterobacteriaceae* and a decrease in *Enterococcaceae*, accompanied by the loss of essential commensal bacteria such as *Alistipes*, *Bacteroides*, *Lachnospira*, and *Barnesiella* [30]. Studies on subjects exposed to the same risk factors have shown a reduction in butyrate production by *Ruminococcaceae*, *Lachnospiraceae*, and *Clostridium* clusters, suggesting that the loss of bacteria that produces beneficial metabolites promotes the onset of CDI [31]. In addition to the well-described decrease in diversity in the CDI group, an underrepresentation of commensal bacteria, including the genera *Alistipes*, *Bacteroides*, *Lachnospira*, and *Barnesiella*, along with the overrepresentation of opportunistic pathogens, such as adherent invasive *Escherichia coli* (AIEC), *Klebsiella pneumoniae*, and *Shigella*, have been observed [32]. CDI relapses progressively worsen dysbiosis, with related metabolic alterations, such as increased primary bile acids and reduced *Clostridiales* and *Collinsella* [33]. Fungal composition is also affected, with an increase in the *Penicillium* genus in affected patients [34]. In summary, CDI induces profound intestinal dysbiosis, characterized by the loss of beneficial commensal bacteria and the expansion of opportunistic pathogens, which contributes to local and systemic inflammation, metabolic and immune dysfunction, and increased susceptibility to relapses and clinical complications.

IBS refers to a chronic functional disorder of the gastrointestinal tract characterized by recurrent abdominal pain or discomfort, bloating, and changes in bowel habits, often accompanied by changes in stool consistency [35]. It can be classified into four subtypes: predominantly constipation (IBS-C), predominantly diarrhea (IBS-D), mixed (IBS-M) and unclassifiable (IBS-U). These subtypes significantly reduce quality of life and can contribute to disability [36]. The overall incidence varies between 10 and 20%, with a constant increase over time. Diagnosis is mainly based on clinical evaluation of symptoms, while instrumental and endoscopic examinations are mainly used to rule out other organic conditions [37]. The pathophysiology of IBS is complex and multifactorial, encompassing alterations in intestinal motility, visceral hypersensitivity, low-grade mucosal inflammation, environmental factors, microbiota imbalances, and psychosocial factors [38]. In recent years, numerous studies have pointed to a central role of intestinal dysbiosis in the genesis and persistence of the disease [39]. In patients with IBS, metagenomic analyses have revealed alterations in microbial diversity and composition. Proteobacteria are often increased, while *Lactobacillus* is reduced, with an overrepresentation of *Ruminococcus gnavus* and some *Lachnospiraceae*. Conversely, *Barnesiella intestinihominis* and *Coprococcus catus* are significantly reduced. These microbial changes can contribute to IBS symptoms, although it is unclear whether they are a cause or a consequence of the disease [40]. Specific patterns arise among the subtypes: IBS-D is characterized by a reduction in *Lactobacillus* and *Bifidobacterium* and an increase in *Enterobacteriaceae*; IBS-C sometimes shows an increase in fecal *Bacteroides* without significant changes in other major genera; IBS-M is associated with a reduction in *Faecalibacterium prausnitzii*, which may increase after treatment with rifaximin; for IBS-U, data are still limited, but an increase in *Pseudomonas aeruginosa* has been noted [41]. Intestinal dysbiosis affects various aspects of intestinal physiology, including the gut-brain axis, intestinal barrier function, nutrient metabolism, and mucosal immune response, suggesting a key role for the microbiota in the onset and chronicity of IBS.

IBD represents a heterogeneous class of chronic disorders with persistent inflammation of the digestive tract, mainly comprising ulcerative colitis (UC) and Crohn’s disease (CD). CD was differentiated from UC in 1932 due to its transmural and segmental inflammatory patterns, affecting any part of the gastrointestinal tract, while UC, which mainly impacts the colon, usually starts in the rectum and progresses to the caecum [42]. Despite advances in technology and experimental models, the etiology of IBD remains mostly unknown. Family studies suggest a genetic component, supported by the discovery of several associated polymorphisms and mutations through genome-wide association studies [43]. However, genetic predisposition alone is rarely sufficient to cause the disease. The increase in the incidence and prevalence of IBD over the last few decades has been too rapid to be explained by genetics alone, suggesting a key role for environmental factors, including lifestyle changes, urbanization and changes in diet, as well as smoking and antibiotic use. In recent years, the focus has moved to microbiota, which under specific conditions can contribute to the initiation and progression of the disease [44]. Studies have shown significant alterations in the gut microbiota in active patients, such as an increase in *Proteobacteria* and a reduction in *Firmicutes*. In genetically predisposed mouse models, the development of colitis is closely dependent on the presence and composition of microbiota, confirming the importance of gut bacteria in IBD pathogenesis. Thus, IBD derives from the interaction of genetic, environmental and microbial factors, each of which is necessary but not sufficient on its own to cause the disease [45]. Although the gut microbiota is generally static over time, changes due to diet, environment, infection or drugs can induce dysbiosis, which in turn affects the host’s immune and metabolic responses. These responses include the production of antimicrobial peptides, reactive oxygen species, immune mediators, and mucosal alterations, resulting in modulation of the composition and function of the microbial community [46]. Studies have shown reduced diversity and an increase in minority phyla such as *Gammaproteobacteria* in patients with IBD, but the mechanistic associations remain largely unknown, often due to a lack of contextual clinical data [47,48]. Most studies have relied upon fecal samples, which only partially reflect regional gut microflora. More recent endoscopic approaches have allowed direct analysis of the intestinal mucosa, revealing significant differences between inflamed and non-inflamed sites and between CD, UC and healthy controls [49,50]. These studies have pointed to the complexity of the gut microbial biogeography, hinting that some alterations can favor the development of pathological states before the onset of clinical symptoms or endoscopic changes [50]. In addition to bacteria, other members of the gut microbiota, such as fungi and viruses, also appear to play a significant role in IBD. Fungal species such as *Candida albicans* and *Malassezia restricta* have been associated with CD, affecting the immune response through pathways such as CARD9 and Dectin-1, the polymorphisms of which are linked to the disease [51]. Viruses, particularly bacteriophages, can also regulate bacterial gene expression and alter the function of the gut microbial community, but their functional role in IBD remains unclear [52].

### 2.2. Factors Modulating the Gut Microbiota

**Figure 1:**
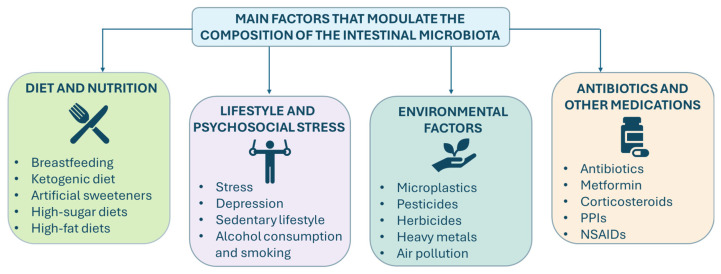
Main extrinsic factors influencing the composition of the intestinal microbiota. Diet and nutrition influence microbial diversity by providing substrates that selectively promote or inhibit specific microbial taxa (e.g., fiber, fats, sugars, artificial sweeteners). Lifestyle and psychosocial factors (e.g., stress, physical inactivity, alcohol consumption, and smoking) impact neuroendocrine and immune pathways, indirectly influencing microbial composition and function. Environmental factors (e.g., MPs, pesticides, heavy metals, and air pollution) can serve as vehicles for intestinal microorganisms or alter host physiological functions, contributing to dysbiosis. Finally, antibiotics and other drugs exert strong selective pressure on the microbiota, often leading to a reduction in diversity. These factors act individually or synergistically to modulate microbial diversity and functionality, ultimately affecting host metabolic, immune, and inflammatory responses.

Various factors, including diet, psychosocial stress, environmental exposures, and the use of antibiotics or other medications, modulate the composition and function of the gut microbiota. These factors often interact synergistically to influence the microbial balance. Indeed, diet, stress, and antibiotic use rarely act in isolation; rather, their combined effects are critical determinants of the composition and function of the gut microbiota. For example, stress influences feeding behaviors, directly modulating microbial composition and, consequently, affecting intestinal motility, immune activation, and barrier function. This bidirectional relationship between stress, diet, and microbiota has been documented in human and animal studies, highlighting their complex interplay in promoting dysbiosis and disease risk. Similarly, antibiotics dramatically alter microbial diversity and ecosystem stability, often reducing the abundance of beneficial taxa and favoring the expansion of opportunistic pathogens [53]. These factors, individually and in synergy, determine dysbiosis, which is associated with numerous chronic conditions, including metabolic disorders, autoimmune diseases, IBD and neuropsychiatric disorders [54]. Understanding the factors that influence gut microbiota composition is essential for developing targeted interventions to maintain or restore gut eubiosis (Figure 1).

#### 2.2.1. Diet and Nutrition

Diet is one of the most influential determinants of gut microbiota composition, with effects observable from early childhood. Infant feeding methods significantly influence early microbial colonization. Numerous studies have shown that breastfeeding promotes the establishment of beneficial microorganisms in the gastrointestinal tract, which are essential for reducing the risk of chronic diseases such as asthma, obesity, allergies, dermatitis, IBD, and neurodevelopmental disorders [55]. In contrast, formula feeding lacks several bioactive components that facilitate optimal microbial colonization [56]. Children breastfed for ≥7 mo have a significantly reduced risk of childhood obesity. A study of 2515 mothers and children aged 2–5 y in Greece found that exclusive breastfeeding for at least 4 mo reduced the risk of childhood overweight and obesity and promoted postpartum maternal weight control [57]. Breastfeeding has also been linked to a greater abundance of beneficial bacteria such as *Lactobacillus* and a reduction in the abundance of potentially pathogenic bacteria, including *Proteobacteria*, *Veillonella*, *Clostridium*, and *Bacteroides* [58]. A study conducted in Qingdao, China, found a significant negative correlation between breastfeeding duration and body mass index in children and adolescents, with children breastfed for more than 12 mo having a lower risk of overweight and obesity [59]. Adolescence is a critical period when the consumption of high-energy processed foods is common and associated with an increased risk of metabolic diseases and alterations in the gut microbiota. A healthy diet promotes a more diverse microbiota with a higher presence of *Bifidobacterium* and *Lactobacillus*, while a high-fat diet increases the anaerobic microflora and the percentage of *Bacteroides*. Similarly, diets high in animal protein enrich bile-tolerant anaerobes such as *Bacteroides*, *Alistipes*, and *Bilophila* [60]. Ketogenic diets, increasingly adopted for rapid weight loss, significantly alter the human gut microbiota, including changes in the relative abundance of *Actinobacteria*, *Bacteroidetes*, and *Firmicutes* [61]. Sweeteners such as acesulfame potassium (Ace-K) and sucralose alter microbial structure and compromise the integrity of the intestinal mucosa [62]. Studies in mice have shown that 8 wk of Ace-K supplementation causes intestinal damage, inflammation, and increased intestinal permeability [63]. Low-dose aspartame increased the abundance of certain bacteria, including *Enterobacteriaceae* and *Clostridium leptum*, while its combination with a high-fat diet further altered the microbial composition and the *Firmicutes*/*Bacteroidetes* ratio [64]. Artificial sweeteners exhibit sex-specific effects: in CD-1 mice, Ace-K for 4 wk increased the abundance of *Bacteroides* in males and altered the abundance of *Anaerostipes* and *Sutterella*, while in females it reduced *Lactobacillus* and *Clostridium* [65].

Combining multiple approaches, such as specific dietary interventions supported by the intake of probiotics and prebiotics, may be more effective in counteracting metabolic syndromes. Lauwn et al. used 16S rRNA sequencing to evaluate the gut microbiota of overweight and obese Hong Kong Chinese subjects before and after an 8-week high-fiber dietary intervention and/or supplementation with two probiotics (*Lactobacillus acidophilus* NCFM and *Bifidobacterium lactis* HN019) and a prebiotic (polydextrose). The study demonstrated that the combined approach was more effective than either approach alone in combating obesity. Specifically, a reduction in fasting blood glucose, insulin secretion, insulin resistance, and triglycerides were observed, while HDL cholesterol levels increased. Furthermore, sequencing data reported a reduction in the number of *Megamonas* in the stool of patients with metabolic syndrome, a bacterial genus positively correlated with body mass index (BMI) and the distribution of total and truncal adiposity [66].

These overall findings indicate the critical role of diet in modulating gut microbiota and highlight how nutrition can exert lasting effects on metabolic health. Importantly, integrated strategies combining dietary interventions with probiotics and prebiotics can improve microbiota composition and metabolic disorders more effectively than individual interventions.

#### 2.2.2. Lifestyle and Psychosocial Stress

Lifestyle factors, particularly psychosocial stress, exert a profound influence on the composition of the gut microbiota through the gut-brain axis, a bidirectional communication network linking the central nervous system (CNS) and the gut microbiota [67]. This axis allows general and host-specific factors, such as stress, depression, and lifestyle, to modulate the microbiota; stress alters microbial composition through hormones, inflammatory cytokines, and the autonomic nervous system [68]. Studies in mice demonstrate that psychosocial stress reduces microbial diversity, decreasing the abundance of beneficial bacteria such as *Bifidobacterium*, *Allobaculum*, *Prevotella*, and *Enterorhabdus*, and increasing potentially pathogenic populations including *Alloprevotella*, *Peptococcus*, and *Anaerotruncus*, resulting in a reduction in the *Actinobacteria*/*Proteobacteria* ratio [69]. Furthermore, stress has been consistently associated with increased abundance of *Euryarchaeota* and decreased *Proteobacteria* in several studies [70,71,72]. In humans, high-stress conditions are correlated with a decrease in *Firmicutes* abundance and an increase in *Bacteroides*, *Rhodococcus*, and *Roseburia* abundance [73]. These observations highlight the susceptibility of the gut microbial ecology to psychosocial stress and suggest that stress management interventions could preserve or restore microbiota balance, supporting both intestinal and systemic health.

#### 2.2.3. Environmental Pollutants

Environmental exposure, particularly to microplastics (MPs), affects the composition of gut microbiota. MPs can alter bacterial enzymatic activity, disrupt microbial homeostasis, and reduce microbial diversity, thereby compromising host immunity, metabolism, and gastrointestinal health [74]. MPs can disrupt the balance of the gut microbiota by acting as a vehicle for specific microorganisms. On these, microbes can form stable biofilms and exploit the associated polymers or contaminants as a carbon source, thus favoring the selection and predominance of certain microbial species in the exposed microbiota [75]. Studies in zebrafish show that exposure to MPs increased the abundance of *Proteobacteria*, stimulated lipopolysaccharide (LPS) production, and promoted intestinal inflammation and damage to the intestinal barrier [76]. Similarly, Chinese crabs exposed to MPs showed a reduction in *Firmicutes* and *Bacteroidetes*, along with the activation of immune-related genes, indicating a pronounced effect on host immune function [77]. These changes collectively suggest that MP-induced microbial changes contribute to dysbiosis, immune dysregulation, and metabolic disorders. Evidence from human studies indicated that MPs reduced microbial diversity and favored pro-inflammatory taxa. The altered balance between *Firmicutes* and *Proteobacteria*, frequently observed following exposure to MPs, is associated with increased susceptibility to metabolic disorders, chronic inflammation, and gastrointestinal diseases, including colorectal cancer [78]. The increasing prevalence of MP contamination in terrestrial and aquatic ecosystems underscores the urgent need to study its impact on the human and animal gut microbiota. Understanding how MPs alter microbiota can guide strategies to reduce exposure and preserve gut balance through diet, probiotics, or plastic pollution regulations.

#### 2.2.4. Antibiotics and Other Medications

Antibiotics and other drugs can exert profound, persisting effects on the gut microbiota. Inappropriate or excessive antibiotic use contributes to antibiotic resistance and alters the microbial balance, with a significant impact on host health [79]. Specific antibiotic regimens have been shown to differentially alter microbial communities. For example, administration of amoxicillin-clavulanate leads to an increase in *Enterobacteriaceae*, including *Enterococci* and *Escherichia coli*, while significantly reducing the populations of beneficial taxa such as *Bifidobacteria*, *Lactobacilli*, and *Clostridia* [80]. In adults, combination therapies containing meropenem, gentamicin, and vancomycin promote the expansion of *Enterobacteriaceae* and other pathogens, accompanied by a decrease in butyrate-producing bacteria [80]. Perinatal antibiotic exposure alters neonatal intestinal colonization patterns, reducing *Actinobacteria* and *Bacteroidetes* and increasing *Proteobacteria* and *Firmicutes*, potentially predisposing infants to immune and metabolic dysregulation in adulthood [81]. In addition to antibiotics, a wide range of commonly prescribed pharmacological agents, including proton pump inhibitors (PPIs), metformin, selective serotonin reuptake inhibitors (SSRIs), nonsteroidal anti-inflammatory drugs, and corticosteroids, have been shown to modulate gut microbial composition [82]. PPIs are associated with increased bacterial colonization of the gastrointestinal tract and altered SCFA production [83]. Metformin alters microbial populations, increasing the relative abundance of *Escherichia coli* and facultative anaerobic bacteria [84]. SSRIs and other psychoactive drugs can affect microbial diversity, such as *Eubacterium ramulus*, while corticosteroids and other immunomodulatory agents affect methanogenic and pro-inflammatory microbial populations, which can influence host metabolism and immune homeostasis [85]. The interaction between pharmacological interventions and the gut microbiota is bidirectional. The microbiota can metabolize drugs, altering their efficacy and toxicity, while drugs can alter microbial composition and function, compromising the intestinal barrier and immune responses. These interactions underscore the importance of considering microbiota-mediated effects when prescribing medications [86]. Restoring the diversity and functionality of the microbiota through dietary interventions, probiotic intake, and appropriate use of antibiotics and other drugs represents a fundamental strategy for maintaining intestinal homeostasis and reducing the risk of disease [87]. Understanding the multifaceted impact of drugs on the intestinal microbial ecology is essential for developing precision medicine approaches that optimize therapeutic outcomes while minimizing adverse effects on the microbiota.

### 2.3. Gut Microbiota and Immunity

Beyond its role in metabolic and dietary processes, the gut microbiota plays a pivotal role in regulating immune responses, particularly through interactions with pattern recognition receptors (PRRs) and other components of the innate immune system. Dysbiosis activates PRRs like Toll-like receptors (TLRs) and NOD-like receptors, stimulating antigen-presenting cells, such as dendritic cells (DCs), which in turn engage T helper (Th) cells and the ensuing adaptive response. This may result in the activation of Th1-, Th2-, or Th17-skewed immunity, depending on the microbial species involved [88]. Th2 dominance, characterized by abnormal production of the cytokines IL-4, IL-5, and IL-13, is associated with the development of IgE-mediated diseases, such as food allergy and asthma [89,90]. Conversely, Th17 dominance is linked to autoimmune and inflammatory conditions, including IBD and rheumatoid arthritis. A host of studies conducted in the last few years have demonstrated the critical interactions of microbiota-delivered signals with FoxP3^+^ T regulatory (Treg) cells, a subset of CD4^+^ T cells centrally involved in immune homeostasis and tolerance. Butyrate and other SCFAs generated upon processing of dietary fibers by such bacterial species as *Faecalibacterium prausnitzii* can support Treg cell development and function via the induction of the vitamin A metabolite retinoic acid, maintaining immune tolerance. Dysbiosis, subsequent to a low-fiber diet, reduces Treg activity, tipping the balance toward effector and inflammatory responses [91]. These responses may in turn weaken the gut barrier, allowing microbial translocation into systemic circulation, which increases systemic inflammation and contributes to metabolic disorders, autoimmunity, and chronic inflammation [92,93]. While host genetics minimally affect the overall microbiota composition, certain immune-related genes, such as those encoding TLRs, may regulate specific microbial groups by shaping relevant immune responses [94]. Therefore, altered TLR signaling can disrupt immune homeostasis, potentially leading to dysbiosis, characterized by an overrepresentation of pathogenic species and a decline in beneficial taxa, often resulting in inflammation [95]. Additionally, microbial balance may be affected by epigenetic changes, including DNA methylation and histone modifications, which regulate mucosal immunity genes [96]. Since dysbiosis contributes to non-communicable diseases, including obesity, diabetes, allergies, asthma, and IBD, by altering immune responses and metabolic regulation, restoring microbial diversity through dietary interventions, probiotics, or reduced antibiotic use may help manage these disorders. This integrated view highlights the profound influence of environmental and epigenetic factors on gut microbiota and immune system dynamics, providing factual evidence in support of the hygiene hypothesis, whereby exposure to declining environmental biodiversity, by adversely affecting the human microbiota and its central functions in immune regulation, would primarily account for the rising prevalence of allergic and other chronic diseases. This underscores the need for lifestyle and therapeutic strategies to restore microbial balance and diversity [97,98].

## 3. Approaches for Studying the Gut Microbiota

**Figure 2:**
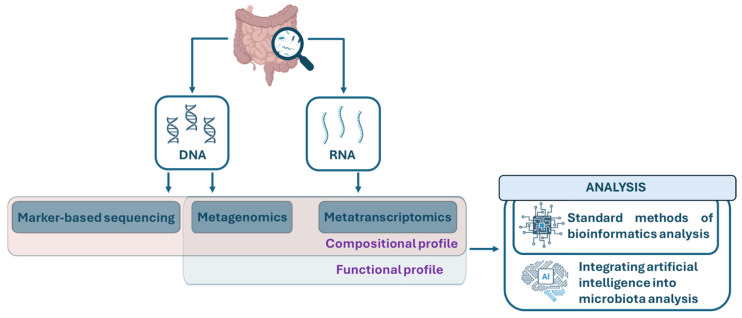
Omics approaches used to characterize the gut microbiota and their integration with bioinformatics analyses and artificial intelligence (AI) tools. Marker-based sequencing (16S rRNA gene) primarily describes the taxonomic composition of the microbiota. Shotgun metagenomics, analyzing total DNA, provides a more in-depth characterization of microbial diversity and the metabolic potential of the community. Metatranscriptomics, based on RNA analysis, allows the study of actual expressed genes, providing insights into the active functionality of the microbiota in specific physiological or pathological conditions. Standard bioinformatics pipelines allow for data preprocessing and statistical analysis, while the application of AI and machine learning algorithms allows for the identification of complex patterns, modeling microbial interactions, and associating microbiota characteristics with clinical phenotypes.

Advances in genomic sequencing have significantly improved the ability to characterize the gut microbiome at high resolution. While early studies focused primarily on bacterial species, current research increasingly includes other microorganisms, such as viruses, fungi, and parasites [99]. Microbiome analysis now relies on a range of sequencing approaches, including amplicon sequencing of the 16S rRNA gene, shotgun metagenomics, and metatranscriptomics [100]. Amplicon sequencing of phylogenetic marker genes, such as the 16S rRNA gene for prokaryotes and the 18S rRNA gene for fungi, allows for the construction of taxonomic profiles. Sequencing reads are filtered to remove technical artifacts and grouped into operational taxonomic units (OTUs) or amplicon sequence variants (ASVs) to increase resolution. Taxonomic assignments are performed using bioinformatics pipelines such as DADA2 or QIIME2, referencing curated databases such as Greengenes and RefSeq [101]. Joo et al. analyzed the gut microbiota of adolescents with gastrointestinal diseases and compared it with that of healthy peers, using 202 fecal samples subjected to 16S rRNA gene sequencing. The study population included adolescents with UC, CD, obesity, and health controls (HC). Data processing was conducted using QIIME2 to generate OTUs and using PICRUSt for functional prediction based on the obtained taxonomic profiles. Subsequently, Kyoto Encyclopedia of Genes and Genomes (KEGG) orthology terms and related functional pathways were analyzed. Microbiota composition showed marked variations in the six most represented taxa, including unclassified *Dorea*, unclassified *Lachnospiraceae*, unclassified *Ruminococcus*, *Faecalibacterium prausnitzii*, *Prevotella copri*, and unclassified *Sutterella*, with differences associated with the presence of inflammatory disease or obesity. Specifically, subjects with UC exhibited increased relative abundances of *Faecalibacterium prausnitzii* (>10%) and unclassified *Lachnospiraceae* (>8%) compared to the CD, obesity, and HC groups. Conversely, obese adolescents had higher levels of *Prevotella copri* (>20%) and unclassified *Sutterella* (>3%) compared to all other groups. The differences reported by Joo et al. are based exclusively on relative abundances, an inherent limitation of 16S rRNA sequencing [102]. This technique does not allow for absolute quantification of taxa, as the number of reads assigned to each microorganism depends on PCR, sequencing depth, and technical biases rather than the actual number of bacteria in the biological sample. The lack of quantitative data limits the biological interpretation of the results, especially in pathological contexts where absolute variations in bacterial load may have diagnostic, prognostic, or pathogenetic significance. In addition to its quantitative limitations, this method cannot detect viruses or eukaryotic microorganisms [103]. These limitations have led to the development of more complex sequencing approaches. Shotgun metagenomics, metatranscriptomics, and advanced computational methods, including AI, have significantly improved microbiome analysis by overcoming the constraints of marker-gene sequencing. Unlike 16S rRNA gene sequencing, these technologies provide both a taxonomic profile and a functional characterization of the microbial community, enabling the simultaneous identification of bacteria, viruses, fungi, and parasites and capturing their functional activity and potential interactions. This integrated approach allows for more comprehensive, quantitative, and mechanistic insights into microbiota, improving the biological interpretation of microbial alterations in health and disease (Figure 2).

### 3.1. Metagenomics

Shotgun metagenomics has established itself as the preferred approach for analyzing microorganisms present in complex environments. This method not only allows comprehensive characterization of the microbial community but also yields absolute quantitative data, overcoming some of the limitations of marker-based sequencing. Continuous technological advances, combined with reduced costs and improved efficiency, have made this technique increasingly accessible, rapid, and economically sustainable [104]. Shotgun metagenomics involves the untargeted sequencing of all the genetic material present in a sample, enabling high-resolution analysis of both the taxonomic composition and functional capabilities of the microbial community [105]. Unlike marker gene-based methods, this approach can detect all microbial constituents, including bacteria, viruses, fungi, and parasites, providing a comprehensive profile of microbiota. An additional advantage is its ability to generate absolute quantitative measurements, providing an accurate estimate of each microorganism’s true abundance [106]. Two main analytical strategies can be applied to shotgun metagenomic data. The first relies on the direct mapping of sequencing reads to genomic databases to determine both the taxonomic structure and functional landscape of the microbiota. Tools such as MetaPhlAn4 enable microbial profiling at the species level, while HUMAnN3 supports the detailed reconstruction of metabolic pathways and functional activities [107]. The second strategy is de novo assembly, in which sequenced DNA fragments are reconstructed into longer contigs, typically using algorithms such as SPAdes or MEGAHIT. These contigs can then be annotated by comparison with reference databases, allowing for a more in-depth exploration of microbial functions, metabolic networks, and ecological interactions. This method also facilitates the identification of previously uncharacterized organisms. However, it is computationally intensive and less effective for organisms at very low abundance [108]. Within this methodological framework, Kim et al. recently applied shotgun metagenomic sequencing to further investigate the possible involvement of the gut-brain axis in the pathophysiology of depressive disorders. The study analyzed the fecal microbiome of a cohort of 133 subjects with depression and 532 nondepressed controls. Through metagenomic profiling, the authors examined the taxonomic structure of microbiota, the biological functions encoded by microbial genomes, and the metabolites potentially derived from these pathways. The analysis revealed microbiota characterized by an overall reduction in the capacity to produce SCFAs, key molecules for intestinal homeostasis and bidirectional communication with the CNS. Specifically, both the metabolic pathways responsible for SCFA biosynthesis and the species known to play this role were depleted in the depressed group. *Faecalibacterium prausnitzii*, a known butyrate producer, was significantly reduced, suggesting a potential biomarker of microbial alterations related to depression. Analysis of the predicted metabolites also suggested a greater abundance in depressed subjects of molecules involved in inflammatory or neuroactive processes, including docosapentaenoic acid, stearoyl ethanolamide, putrescine, and bilirubin [109]. These overall results highlight how the shotgun metagenomic approach represents a more powerful and informative tool than marker-gene-based sequencing. While the latter provides a limited view of the microbial community, metagenomics offers a comprehensive characterization of the microbiota, allowing the simultaneous identification of bacteria, viruses, fungi, and parasites, the reconstruction of their biological functions, and the absolute quantification of their abundance [110]. This level of detail is essential to fully understand the microbial alterations associated with complex pathologies.

### 3.2. Metatranscriptomics

Metatranscriptomics has emerged as a complementary technology that provides dynamic insights into microbiome functions. Unlike DNA-based methods, metatranscriptomics sequences RNA at the community level, capturing gene expression in real time and allowing researchers to distinguish between metabolically active and inactive microbes. This approach reveals transcriptionally regulated pathways, host-microbe interactions, and functional changes that may not be detectable at the genomic level [111]. When combined with metagenomic data, metatranscriptomics enables a comprehensive multi-omics understanding of the microbiome, linking microbial potential (genes) with microbial activity (transcripts). A clear demonstration of the added value of metatranscriptomics comes from the work of Dora et al., who studied the relationship between gut microbiome activity and the response to immune checkpoint inhibitors (ICIs) in advanced non-small cell lung cancer. In this study, fecal samples from 29 patients treated with ICIs were analyzed using a de novo metatranscriptomic assembly strategy. Patients were stratified based on progression-free survival (PFS) into patients with long (>6 mo) and short (≤6 mo) responses. Using an RNA-seq workflow based on Trinity for assembly, MMSeqs2 for taxonomic classification, and DESeq2 for differential expression analysis, the authors coupled microbial functional signatures with machine learning (ML) models, including Random Forest, Support Vector Machine, and XGBoost algorithms. Although alpha diversity did not differ significantly between groups, beta diversity analysis revealed a clear separation between patients with long and short responses. *Actinomycetota* and *Euryarchaeota* were significantly enriched in patients with short PFS, while *Bacillota* predominated in individuals with long PFS. Differential expression analysis identified 120 transcripts that could distinguish the two groups: gene clusters associated with DNA synthesis and translesional repair were upregulated in patients with short PFS, while metabolic pathways, largely driven by *Escherichia coli*-derived PFAM domains, were more active in patients with long PFS. Importantly, all ML models confirmed the predictive value of this RNA-based microbial signature (ROC AUC > 0.84), and multivariate Cox regression, incorporating PD-L1 expression and chemotherapy history, highlighted six metatranscriptomic biomarkers independently associated with PFS [112]. Overall, these findings demonstrate how metatranscriptomics overcomes the limitations of gene-marker sequencing by capturing the functional state of the microbiome rather than simply its taxonomic composition. While 16S rRNA or other marker gene-based approaches provide valuable but static snapshots of microbial communities, metagenomic and, especially, metatranscriptomic sequencing offer a more comprehensive and mechanistic understanding of how microbial functions interact with host physiology and therapeutic response. In this context, metagenomics and metatranscriptomics emerge as superior tools, enabling a more in-depth and accurate characterization of the microbiome-mediated mechanisms underlying clinical outcomes [113].

### 3.3. Artificial Intelligence

Recent developments in advanced computational methodologies and AI applications have profoundly transformed gut microbiota research, enabling the data generated by the sequencing approaches to be more effectively exploited. These tools now allow the identification of key biomarkers useful for the diagnosis, prognosis, and therapeutic management of numerous diseases, integrating complex information on the composition, function, and interactions of the microbiota with the host [114]. Recent advances in computational methodologies and AI applications have revolutionized the study of gut microbiota, enabling the identification of key biomarkers for the diagnosis, prognosis, and therapeutic management of various diseases [115]. AI has overcome the limitations of traditional microbial analysis methods, offering tools capable of rapidly analyzing large volumes of complex data. Thanks to ML and deep learning (DL) techniques, it is now possible to recognize low-abundance pathogens [116]. In the context of the gut microbiome, ML has proven particularly useful in analyzing high-dimensional datasets, enabling the selection of biomarkers, the prediction of clinical phenotypes, and the classification of samples [117]. DL applications have further expanded analytical possibilities, for example, predicting antibiotic resistance or modeling microbial interactions over time. More advanced techniques, such as graph neural networks, allow the reconstruction of networks of co-occurrence and complex functional relationships between microorganisms [118]. These approaches are particularly effective when integrated with multi-omics, metagenomics, metatranscriptomics, metabolomics, and proteomics data, providing a comprehensive view of the structure, function, and interaction of the microbiota with host health [119]. Overall, AI and ML are redefining gut microbiome research, improving the interpretation of large omics datasets, facilitating the identification of diagnostic and prognostic biomarkers, and enabling the development of predictive models essential for precision medicine [120]. Liu et al. reported that analyzing the gut microbiota using ML could represent a noninvasive strategy for diagnosing liver cirrhosis and fibrosis. To further explore this topic, the authors conducted the first systematic review and meta-analysis to evaluate the predictive accuracy of gut microbiota-based models. From a systematic search of PubMed, the Cochrane Library, Embase, and Web of Science, updated to 2 April 2023, 10 studies were selected, comprising 11 prediction cohorts and 838 participants, including 403 patients with liver fibrosis or cirrhosis. The meta-analysis showed overall sensitivity values of 0.81 [0.75–0.85], specificity 0.85 [0.77–0.91], positive likelihood ratio 5.5 [3.6–8.7], negative likelihood ratio 0.23 [0.18–0.29], diagnostic odds ratio 24 [14,15,16,17,18,19,20,21,22,23,24,25,26,27,28,29,30,31,32,33,34,35,36,37,38,39,40,41], and area under the ROC curve 0.86 [0.83–0.89]. These results indicate that ML models applied to gut microbiota data have high predictive capacity for cirrhosis and liver fibrosis, offering a noninvasive diagnostic approach with broad clinical potential [121]. Wu et al. highlighted the crucial role of AI and ML in identifying diagnostic biomarkers based on the gut microbiota. In the study, 300 biomarkers were selected from a total of 13,990 features, including clinical information and the relative abundance matrix of genes, derived from 806 microbiomes of Chinese individuals (383 controls, 170 with type 2 diabetes, 130 with rheumatoid arthritis, and 123 with liver cirrhosis). Seven ML algorithms were tested, with logistic regression achieving the highest accuracy. The developed pipeline generated an F1 score of 0.91 and an area under the ROC curve of 0.95, confirming the strong correlation between the selected biomarkers and the observed phenotypes. The identified biomarkers demonstrated a high capacity to distinguish healthy individuals from patients with diseases, highlighting how AI can transform microbiome analysis from a simple descriptive characterization to a highly accurate predictive tool, applicable to large-scale diagnostics and screening. The study also highlighted specific characteristics of disease phenotypes: in patients with type 2 diabetes, an altered *Bacteroides*/*Firmicutes* ratio was observed, consistent with previous studies; in patients with rheumatoid arthritis, biomarkers were depleted in Gram-negative bacteria and enriched in Gram-positive bacteria; while in liver cirrhosis, genera such as *Streptococcus*, *Veillonella*, and *Faecalibacterium* were significantly enriched [121]. The integration of clinical information and microbiome profiles using AI algorithms enables precise prediction of individual health status, underscoring the potential of ML not only for biomarker discovery but also for the development of microbiome-based personalized medicine strategies [122]. In conclusion, advances in genomic sequencing and multi-omics technologies, combined with the application of AI and automated algorithms, have profoundly transformed the study of the gut microbiome. The choice of ML models is guided by their ability to adapt to the nature, dimensionality, and heterogeneity of microbiota data, while the use of multiple approaches allows for cross-validation of results, reduction in bias, and optimization of the tradeoff between predictive accuracy and interpretability. Integrating these tools with AI approaches amplifies their analytical potential, enabling deeper data analysis, the identification of hidden relationships, and the development of predictive models to support precision medicine.

## 4. Emerging Strategies for Restoring Gut Microbiota Balance

**Figure 3:**
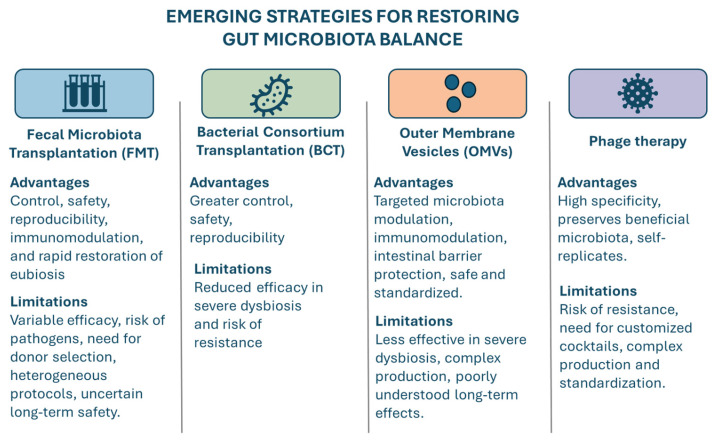
Overview of emerging strategies to restore gut microbiota balance. Four main approaches—FMT, BCT, OMVs, and phage therapy—offer distinct advantages despite specific limitations. FMT rapidly restores eubiosis and modulates immunity but requires careful donor selection and carries risks of variable efficacy and pathogen transfer. BCT provides standardized, controlled bacterial interventions but may be less effective in severe dysbiosis. OMVs enable targeted microbiota modulation and immune support while avoiding live bacteria, though production is complex and long-term effects are not fully understood. Phage therapy offers high specificity and self-replicating antimicrobial activity, yet it demands customized cocktails and faces challenges in standardization and resistance management.

Maintaining a balanced gut microbiota is crucial for overall health, as alterations in this ecosystem can disrupt host physiology and immune homeostasis [123]. Therapeutic strategies to restore intestinal eubiosis include probiotics, which outcompete harmful microbes; prebiotics, which promote the growth of beneficial bacteria; and symbiotic, which combine both approaches for greater efficacy [124]. Emerging therapies are expanding the scope of microbiota-targeted interventions, including FMT, bacterial consortium transplantation (BCT), phage therapy, and application of OMVs. Although these approaches differ in their mechanisms, they share a common goal of replacing pathogenic or dysbiotic microbes with beneficial ones, thereby restoring gut eubiosis [125] (Figure 3).

### 4.1. Fecal Microbiota Transplantation (FMT)

FMT involves the transfer of a healthy donor’s microbiota to a recipient to restore gut eubiosis. It is being studied for a wide range of conditions, including gastrointestinal disorders, psychiatric conditions, skin diseases, and metabolic disorders. FMT initially attracted attention for the treatment of recurrent CDI, IBD, and IBS, with ongoing trials exploring its potential for other conditions [126]. FMT can be conceptualized as a microbiota-organ transplant, in which the microbial community of a healthy donor is transferred to a recipient to restore intestinal eubiosis [127]. Globally, several clinical trials are currently investigating FMT as a potential treatment for a wide range of conditions, including gastrointestinal disorders, psychiatric and neurodevelopmental conditions, skin diseases, metabolic disorders, antibiotic-resistant bacterial infections, cancers, connective tissue diseases, and neurodegenerative disorders. FMT initially gained prominence in the treatment of intestinal diseases, and this area remains a primary focus of research [128]. FMT is a proven treatment for antibiotic-resistant *Clostridium difficile* diarrhea. The Food and Drug Administration (FDA) approved RBX2660 (Rebyota) in November 2022 as the first commercial FMT product to prevent recurrent infections in adults. Derived from donor stool, it contains key bacteria such as *Bacteroides*, which play a role in resistance to *Clostridium difficile* colonization. A Phase III study demonstrated that RBX2660 had a higher success rate (70.6%) than placebo (57.5%) in preventing recurrence [129]. FMT rapidly alters the recipient’s fecal microbiota to resemble that of a healthy donor, with these changes lasting at least 24 wk. The procedure requires careful donor selection, including comprehensive blood and stool screening for infectious diseases. Additionally, the donor’s microbiota must be assessed for eubiosis, either by quantitative PCR or next-generation sequencing, to ensure safety and efficacy. Selecting an unsuitable donor could pose significant health risks, such as nutritional imbalances or chronic conditions. To improve convenience and patient experience, capsule administration of fresh bacterial preparations is being explored as a more practical alternative to traditional FMT methods [130,131]. Moayyedi et al. conducted a randomized, placebo-controlled, parallel study to evaluate the safety and efficacy of FMT in patients with active UC, excluding subjects with infectious diarrhea. Participants underwent flexible sigmoidoscopy at study entry and were randomized to receive weekly 50 mL enemas of FMT from healthy anonymous donors (n = 38) or placebo water enemas (n = 37) for a period of 6 weeks. The study was conducted in a patient-, clinician-, and investigator-blinded fashion. The primary endpoint was clinical and endoscopic remission at wk 7, defined as a Mayo score ≤ 2 and an endoscopic Mayo score = 0. Remission was observed in 24% of subjects treated with FMT compared to 5% of the placebo group, for a statistically significant risk difference of 17% (95% CI: 2–33%). No significant differences in adverse events were found between groups. Most remissions (7 out of 9) were achieved using fecal material from a single donor. Furthermore, disease duration appeared to influence outcome, with higher remission rates in patients with UC ≤ 1 y compared to those with UC > 1 y (3/4 vs. 6/34; *p* = 0.04). Fecal microbiome analysis showed an increase in microbial diversity in subjects treated with FMT compared to baseline (*p* = 0.02), while no significant change was observed in the placebo group. These data support the role of FMT as a safe and effective strategy for inducing clinical and endoscopic remission in patients with active UC, highlighting the importance of donor selection and timing of the intervention. Modulation of the gut microbiota through FMT appears to correlate with clinical remission, suggesting an immunomodulatory and protective mechanism mediated by the reconstitution of gut microbial diversity [132]. Similarly, Rossen et al. evaluated the efficacy and safety of FMT in patients with mild to moderate UC using a randomized, controlled, double-blind study. Fifty patients were assigned to receive FMT from healthy donors or autologous microbiota (control), administered via nasoduodenal tube at baseline and 3 wk later. The primary endpoint combined clinical remission, defined as a Simple Clinical Colitis Activity Index ≤ 2, with a reduction of at least 1 point in the Mayo endoscopic score at 12 wk. Secondary endpoints included safety and fecal microbiota composition, assessed by phylogenetic microarray. Thirty-seven patients completed the primary endpoint assessment. In the intention-to-treat analysis, 30.4% of patients treated with FMT from healthy donors achieved remission compared to 20% of controls, while in the per-protocol analysis, remission occurred in 41.2% of the FMT group and 25% of the control group. The data are not statistically significant. Four patients experienced serious adverse events, two of whom were in the FMT group, which were not attributable to the treatment. Microbiome analysis at 12 wk showed that responders in the FMT group had a microbiota similar to that of healthy donors, with a greater abundance of *Clostridium* clusters IV and XIVa, suggesting a possible correlation between microbial composition and clinical response. Despite the lack of statistically significant differences between the groups, likely due to the limited sample size, the study highlights that response to FMT may be associated with specific characteristics of the microbiota. These results highlighted the importance of further investigating the relationship between microbial composition and clinical outcomes in order to identify optimal donor profiles and improve the efficacy of FMT in UC [133]. Furthermore, Crothers et al. evaluated long-term FMT in patients with UC using orally administered encapsulated frozen stool (cFMT). In this randomized study, subjects were assigned 1:1 to receive FMT induction via colonoscopy, followed by 12 wk of daily administration of cFMT or oral placebo (sham therapy). Follow-up lasted 36 wk, with longitudinal clinical assessments based on subjective and objective parameters of disease severity. Fecal microbiota analysis was performed by 16S rRNA sequencing, while changes in Treg cell and mucosal-associated invariant T (MAIT) cell populations were assessed by flow cytometry as an exploratory endpoint. Twelve patients with active UC were randomized; six completed the full course of cFMT and six received placebo treatment. Chronic administration of cFMT was shown to be safe and well tolerated, although concerns regarding home storage arose. Adherence to the protocol was high, and no FMT-related adverse events were reported. Two patients treated with cFMT achieved clinical remission, while no patients in the placebo group achieved remission (95% CI = 0.38–∞, data not significant). cFMT induced sustained changes in fecal microbiota composition, associated with changes in cytokine production by MAIT cells, which correlated with clinical response. These preliminary data indicate that daily administration of cFMT may prolong the persistence of FMT-induced changes in the intestinal microbial ecosystem. Furthermore, the association between MAIT cell activity and clinical response suggests a possible immunomodulatory mechanism related to microbiota modulation [134]. Minkoff et al. evaluated the efficacy of FMT in the treatment of recurrent CDI (rCDI). Because standard antibiotic treatment can further exacerbate intestinal dysbiosis, predisposing to recurrence, FMT has emerged as a promising approach aimed at restoring a healthy microbial ecosystem. In a systematic review and meta-analysis conducted using Cochrane methodology, the authors assessed the benefits and potential risks of FMT in immunocompetent subjects with rCDI, including six randomized trials with a total of 320 participants. The studies, conducted in Europe and North America, used various administration routes (nasoduodenal, naso- or nasojejunal, enema, or colonoscopy), while control groups included placebo, autologous FMT, no treatment, or antibiotics (mainly vancomycin). The combined results show that FMT likely significantly increases the resolution of rCDI compared to comparator treatments (RR = 1.92; 95% CI: 1.36–2.71; NNT = 3; moderate-certainty evidence). A possible reduction in serious adverse events was also observed (RR = 0.73; 95% CI: 0.38–1.41), although with wide confidence intervals that preclude definitive conclusions. A favorable trend also emerged for all-cause mortality, but the low number of events limits the strength of the evidence. None of the studies reported colectomy-related outcomes. The authors conclude that, in immunocompetent adults, FMT represents a highly effective treatment for rCDI compared to conventional therapies, with a generally favorable safety profile. However, the paucity of reported serious adverse events and the lack of large, long-term studies warrant further research, ideally supported by national registries. Furthermore, the small number of immunocompromised participants does not allow us to extend these conclusions to this population, for which dedicated studies remain necessary [135]. The response to FMT varies depending on the pathology being considered. Osaki et al. compared the efficacy of FMT in patients with UC, CD, and rCDI. The study involved 42 patients, who received stool solution endoscopically. FMT performance was assessed 8 wk after transplantation using the Mayo score for UC, the CDAI for CD, and the absence of toxin-negative diarrhea in rCDI patients. Additionally, the fecal microbiota was analyzed using 16S ribosomal RNA sequencing and by assessing fecal SCFA levels. Clinical response was observed in 25% of UC patients, 75% of CD cases, and all rCDI patients. Clinical remission was observed in 20% and 25% of UC and CD cases, respectively. Specifically, UC patients had a reduced *Clostridium* cluster XIVa load before FMT, followed by an increase after treatment. Furthermore, a higher abundance of *Fusicatenibacter saccharivorans* in donors was associated with clinical remission at 8 wk. In CD cases, lower abundances of *Blautia*, *Dorea*, and *Eubacterium* were detected before FMT, while after treatment, an increase in *Collinsella*, *Dorea*, and *Eubacterium* was observed, with increased fermentation of SCFAs and elevated fecal butyrate concentrations. Patients with rCDI showed significantly reduced levels of *Clostridium* clusters IV and XIVa before transplantation, which increased after treatment, in association with elevated fecal propionate concentrations. Overall, this study showed greater efficacy of FMT in rCDI, intermediate efficacy in CD, and more limited efficacy in UC, at least in the context of this study [136].

Meta-analyses involving larger populations indicate that FMT is effective in promoting clinical and/or microbiological remission in both patients with UC and rCD. Feng et al. conducted a meta-analysis using 13 randomized controlled studies involving a total of 580 UC patients (293 treated with FMT and 287 controls), confirming that FMT significantly increased clinical remission compared to controls (RR 1.73; 95% CI 1.41–2.12; *p* < 0.00001) and also improved endoscopic remission (RR 1.74; 95% CI 1.24–2.44; *p* = 0.001). This analysis did not show a significant increase in adverse events among patients treated with FMT compared to controls (RR 1.00; 95% CI 0.86–1.15; *p* = 0.96), suggesting a good safety profile. However, maintaining long-term endoscopic remission remains a challenge, underscoring the need for further studies to optimize donor selection, dosing, administration route, and follow-up strategies [137]. Furthermore, in a meta-analysis of 15 studies involving 777 rCDI patients, Porcari et al. evaluated the efficacy and safety of FMT following at least 8 wk of follow-up. The analysis showed 81% and 92% cure rates for single FMT and overall FMT, respectively (based on nine studies with 354 patients) (*p* = 0.0015). Serious adverse events occurred in 91 patients (12%). These data indicate that FMT is highly effective in treating rCDI even in patients with IBD [138].

Overall, the available evidence confirms that FMT represents an innovative and extremely promising therapeutic strategy for numerous conditions mediated or accompanied by intestinal dysbiosis. The main advantages of FMT include the ability to rapidly reestablish a eubiotic microbial community, the possibility of achieving clinical remissions in patients refractory to conventional treatments, its immunomodulatory potential, and the growing availability of standardized and regulated formulations, which improve safety, traceability, and accessibility. Furthermore, the adoption of non-invasive approaches, such as oral capsules, is helping to make the procedure more acceptable and easily implementable [139]. From a cost–benefit perspective, FMT is generally associated with low initial production costs due to its use of donor-derived material and relatively simple processing procedures. However, these economic advantages are partially offset by indirect costs related to donor screening, testing for emerging pathogens, product variability, and the need for invasive administration methods (e.g., colonoscopy or nasoenteric infusion), which increase procedural costs and healthcare resource utilization. Furthermore, variability in clinical response may require multiple administrations, further increasing overall treatment costs [140,141]. Furthermore, uncertainties persist regarding long-term safety, particularly regarding the transmission of undetected pathogens, potential adverse metabolic or immunological effects, and poorly defined risks for immunocompromised individuals. Finally, the lack of international standardization and the limited availability of large, well-controlled studies on certain extraintestinal indications require further investigation before FMT can be considered an established therapy outside of its current primary applications [142] (Table 1).

### 4.2. Bacterial Consortium Transplantation (BCT)

BCT is a promising alternative to FMT to modulate the gut microbiota, particularly in the treatment of CDI. Studies show that BCT can restore the balance of the gut microbiota after antibiotic-induced dysbiosis, providing comparable benefits to FMT [143]. Petrof et al. demonstrated that a synthetic bacterial consortium (BC) derived from stools from healthy donors can effectively treat CDIs, representing a potential alternative to traditional FMT. The consortium, including strains such as *Bacteroides ovatus*, *Bifidobacterium lingua*, and *Lactobacillus casei*, was developed as a stool substitute from purified intestinal bacterial cultures from a single healthy donor (clinical-stage product). This approach addresses the main limitations of conventional FMT, such as the risk of donor-derived infection and poor patient acceptance. In the pilot study, two patients with recurrent CDI who had failed at least three courses of standard antibiotics (metronidazole or vancomycin) were treated by infusing the bacterial BC through colonoscopy, extending the administration to the right and midcolon. Both patients were infected with hypervirulent strains of *Clostridium difficile* (ribotype 078). After treatment, patients restored a normal intestinal pattern within 2–3 days and remained asymptomatic for 6 months. Analysis of the fecal microbiota by 16S rRNA sequencing showed that species present in the consortium, rare in the pre-treatment microbiota, constituted more than 25% of the sequences up to 6 mo after intervention, indicating effective colonization and persistence of the therapeutic bacteria. These results constitute a proof of principle that a purified multi-species bacterial mixture can resolve antibiotic-resistant infection, associated with significant changes in fecal microbial composition. This strategy paves the way for more standardized and safer interventions than conventional FMT, with potential applicability in other infectious gastrointestinal diseases [144]. Similarly, Quaranta et al. proposed the use of a synthetic bacterial suspension, composed of 13 microbial species isolated through culturomics protocols from the stool of healthy donors. These include *Acidaminococcus intestini*, *Bacteroides fragilis*, *Bacteroides ovatus*, *Bacteroides uniformis*, *Bifidobacterium longum*, *Clostridium scindens*, *Lactobacillus casei*, *Lactobacillus gasseri*, *Lactobacillus rhamnosus*, *Lactobacillus parabuchneri*, *Parabacteroides distasonis*, *Propionibacterium avidum*, and *Ruminococcus gnavus*, with a count of 5 × 10^8^ cfu/mL (clinical-stage product). The efficacy of the treatment was evaluated both clinically and by metagenomic typing. Patients’ stool samples were collected before and after the infusion, and the extracted DNA was analyzed by next-generation sequencing at various time points (pre-infusion, 7, 15, 30, and 90 d post-infusion). In patient 1, the pre-infusion gut microbiota was dominated by *Bacteroidetes*; 7 d after infusion, a decrease in *Bacteroidetes* was observed, accompanied by an increase in *Firmicutes* and *Verrucomicrobia*. Patient 2 had, before treatment, a predominance of *Proteobacteria* and a significant deficiency in *Bacteroidetes* and *Verrucomicrobia*; 7 d after infusion, *Proteobacteria* decreased markedly, while *Bacteroidetes* and *Verrucomicrobia* increased. Metagenomic analysis also highlighted a true “activation” of microbial species absent or poorly represented at time T0 but present after infusion. These results suggest that the infusion of selected bacteria can act as a trigger for bacterial repopulation, representing an innovative therapeutic approach for the treatment of bacterial infections such as CDI [145]. In April 2023, the FDA approved SER-109 (Vowst), the first oral *Firmicutes* spore-based treatment (bacteriotherapy capsule therapy) for the prevention of rCDI in adults. This formulation is designed to selectively restore depleted *Firmicutes* populations after antibiotic treatment, significantly reducing relapse rates. Unlike traditional broad-spectrum fecal transplants, Vowst offers a more controlled and standardized approach, ensuring the administration of a defined set of bacteria, excluding viruses and unwanted pathogens, and improving the overall safety of the treatment. Side effects reported in pivotal clinical trials included gastrointestinal upset, bloating, and fatigue, generally mild or moderate. The mechanisms by which SER-109 prevents rCDI include direct competition with *Clostridium difficile*, bacteriocin production, and modulation of the intestinal microbiota, with an increase in beneficial bacteria such as *Bacteroidetes*, *Clostridium* clusters IV and XIVa, *Fecalibacterium prausnitzii*, *Butyrivibrio crossotus*, *Enterococcus*, *Lactobacillus*, and *Veillonella*, while simultaneously reducing pathogenic bacteria. The viromic composition of the donor, particularly the presence of *Caudovirales*, appears to contribute to improving the efficacy of the therapy. The ECOSPOR III and IV clinical studies confirmed both the safety and efficacy of SER-109. In ECOSPOR III, a double-blind, randomized study, SER-109 significantly reduced the risk of rCDI (RR = 0.32; 95% CI: 0.18–0.58) compared to placebo at 8 wk, with effects maintained for up to 24 wk. *Firmicutes* spore engraftment was associated with the formation of secondary bile acids, which are bacteriostatic for *Clostridium difficile*, contributing to a durable clinical response. Adverse events were primarily mild or moderate, with no serious events attributable to treatment. Secondary analyses showed a significant improvement in quality of life as early as 1 wk and continued up to 8 wk, while the use of SER-109 contributed to a reduced burden on the healthcare system, with fewer hospitalizations and emergency room visits due to rCDI. Overall, these data support SER-109 as an innovative, safe, and standardized approach for the prevention of rCDI, with clear advantages over traditional fecal transplants. However, open questions remain, including its efficacy in specific populations such as pregnant or breastfeeding women, cancer patients, or immunocompromised patients, as well as the need for further large-scale cost-effectiveness studies [146]. McGovern et al. explored the efficacy of SER-109 in an elderly population (n = 89) with rCDI. SER-109 reduced rCDI rates compared to placebo in subjects ≥ 65 years of age. Specifically, among the subjects treated with BCT, only 45.2% showed rCDI, while in the placebo group, 80% of subjects showed the disease [147].

While BCT is associated with higher development and production costs due to strain isolation, characterization, manufacturing, and quality control, it offers clear advantages over FMT, including better control, safety and reproducibility. BCT uses stable and standardized bacterial strains, avoiding logistical problems associated with fecal material. Increased standardization, scalability, and reproducibility translate into more predictable clinical outcomes, simplified regulatory pathways, and reduced long-term costs associated with safety monitoring and adverse event management. Furthermore, oral BCT formulations can significantly reduce administration costs.

However, its efficacy can be affected by the competitive ability of introduced strains and the risk of acquiring harmful traits such as antibiotic resistance. BCT may also be less effective in patients with severe dysbiosis, and optimizing bacterial strains for competition requires extensive research. These challenges underscore the need for continued research and caution in the clinical application of BCT [148,149] (Table 2).

### 4.3. Outer Membrane Vesicles (OMVs)

OMVs are nanosized particles released by Gram-negative bacteria, including those of the gut microbiota. They play a crucial role in maintaining intestinal eubiosis, the balanced state of the gut microbiota, by facilitating interbacterial communication, modulating host immune responses, and influencing intestinal epithelial cell function. OMVs contain a variety of bioactive molecules, such as lipids, proteins, and nucleic acids, which can interact with host cells and other microbes. This interaction helps maintain the integrity of the intestinal barrier and supports a healthy microbiota composition [150]. Research indicates that OMVs can modulate the intestinal microbiota by promoting the growth of beneficial bacteria and suppressing pathogenic bacteria. Zakharzhevskaya et al. demonstrated that OMVs derived from *Bacteroides fragilis* play a crucial role in intestinal homeostasis and immune response modulation. OMVs from non-toxigenic Bacteroides fragilis (NTBF) not only support the nutritional needs of other microorganisms but also exert anti-inflammatory effects on immune cells, highlighting their beneficial role in the gut. In contrast, toxigenic Bacteroides fragilis (ETBF) is associated with intestinal diseases, including colorectal cancer, suggesting that ETBF-derived OMVs may have pathogenic potential. To explore these differences, a comparative proteomic analysis of ETBF and NTBF OMVs was conducted by HPLC-MS/MS, complemented by metabolomic profiling by HPLC-MS/MS and GC-MS, and the reconstruction of the related metabolic pathways; fluxomics experiments validated the reconstructed biochemical activities. Analyses revealed marked proteomic differences: ETBF OMVs contain a broader and more diverse set of proteins than NTBF OMVs (392 vs. 291 in NTBF), including unique periplasmic proteins and many cytoplasmic proteins involved in transcription, DNA replication, translation, and stress response, which are absent in NTBF OMVs. In contrast, NTBF OMVs exhibit a simpler cytoplasmic protein profile, with only a few translation-related proteins. Both strains contain clostripain, which is inactive in NTBF due to mutation. These differences suggest that ETBF OMVs act as functional, potentially pathogenic microreactors, while NTBF OMVs reflect limited, nonpathogenic functionality. Metabolomic analysis revealed significant differences in the content of 95 metabolites in bacterial cells and in ETBF and NTBF OMVs, including compounds involved in amino acid and nucleotide catabolism, glycolysis, the TCA cycle, and cofactors. OMVs contained oxidized and reduced forms of free fatty acids; in particular, NTBF OMVs showed increases in six essential amino acids, creatine, creatinine, D-glycerate-2-phosphate, fumarate, malate, FAD, and GMP, indicating partial metabolic activity of glycolysis and purine nucleotide catabolism. In ETBF OMVs, the decrease in TCA and FAD intermediates suggests a more active TCA cycle. The metabolic correlation between cells and OMVs was weak, and some metabolites were exclusive to the vesicles (e.g., L-histidine, mannitol, nicotinic acid, sucrose, xanthosine, D-fructose-1,6-PP, trehalose), while cytidine and gluconate were present only in NTBF OMVs. Overall, the metabolic profile of the cells was similar to that of the respective OMVs, although they displayed metabolic species specific to ETBF or NTBF. These results provide a mechanistic basis for the dual role of B. fragilis OMVs in intestinal homeostasis and disease, offering new insights into host-microbiota interactions and their potential as a therapeutic target [151]. In addition, Wang et al. highlighted the functional significance of OMVs released by *Akkermansia muciniphila* in maintaining intestinal homeostasis. These vesicles contribute to intestinal health through multiple mechanisms. They restored dysbiotic microbiota by selectively promoting the growth of beneficial bacteria through membrane fusion. They enhanced mucosal immunity by translocating into Peyer’s patches and activating B cells and DCs, thereby inducing IgA secretory responses. Furthermore, they preserved the integrity of the intestinal barrier, upregulating tight junction proteins and mucus production. Importantly, in vivo administration of *Akkermansia muciniphila* OMVs alleviated colitis and improved the efficacy of anti-programmed cell death protein 1 (PD-1) immunotherapy in colorectal cancer models, highlighting their role in modulating host-microbiota interactions. These findings highlight OMVs as critical effectors in intestinal ecology and suggest their potential as therapeutic targets for modulating microbiota composition and treating gastrointestinal disorders [152]. Kang et al. highlighted the anti-inflammatory potential of extracellular vesicles (EVs) derived from *Lactobacillus kefirgranum* PRCC-1301 (PRCC-1301 EVs) in modulating intestinal inflammation and maintaining epithelial barrier function. In vitro studies, human intestinal Caco-2 cells treated with PRCC-1301 EVs and subsequently stimulated with dextran sulfate sodium (DSS) showed a significant reduction in the expression of pro-inflammatory cytokines, as detected by quantitative RT-PCR. Furthermore, EVs preserved the integrity of epithelial cells and the tight junction complex, restoring the levels of Zo-1, claudin-1, and occludin in both Caco-2 cells and colonic tissues, as demonstrated by immunofluorescence analysis. In murine models, acute colitis was induced with 4% DSS, while chronic colitis was generated in IL-10^−/−^ mice treated with piroxicam. Treatment with PRCC-1301 EVs attenuated body weight loss, colonic shortening, and histological damage in both models. Immunohistochemical analysis also revealed decreased levels of phosphorylated NF-κB p65 and IκBα in colonic tissues. These data suggest that PRCC-1301 EVs exert protective effects on the intestine by modulating the inflammatory response through inhibition of the NF-κB pathway and contributing to the maintenance of intestinal barrier integrity, confirming their therapeutic potential in colitis [153]. In the study by Ma et al., the mechanisms by which *Clostridium butyricum* EVs contribute to the improvement of IBD were investigated using a mouse model of DSS-induced colitis. Administration of *Clostridium butyricum*-derived EVs attenuated colonic barrier disruption and modulated inflammatory responses. Transcriptomic analysis (RNA-seq) revealed that EVs significantly regulated genes involved in inflammation and immune pathways. KEGG enrichment highlighted modulation of IL-17, TNF-α, MAPK, PI3K-Akt, cytokine-cytokine receptor interactions, and NF-κB signaling, with EVs also influencing mucin-type O-glycan biosynthesis, purine metabolism, and FoxO signaling. Gene ontology analysis confirmed their involvement in inflammatory regulation, ERK1/2 cascades, and immune modulation. Specifically, EVs demonstrated a greater ability than whole bacteria to mitigate DSS-induced upregulation of genes associated with inflammation and tumor pathways. EVs also reshaped the gut microbiota, increasing its diversity and altering its structure. EVs promoted the proliferation of genera including *Ruminiclostridium*, *Anaerofustis*, *Helicobacter*, and *Alistipes*, highlighting the unique and potentially complementary role of microbial EVs in modulating the gut microbial ecology and alleviating colitis [154]. Fábrega et al. used a mouse model of DSS-induced colitis to evaluate the therapeutic potential of OMVs produced by *Escherichia coli* Nissle 1917 (EcN) in modulating mucosal damage and intestinal inflammation. The experimental protocol involved oral pretreatment with purified OMVs (5 μg/d) for 10 d, followed by colitis induction via DSS and a 5-day recovery period [155]. This strategy resulted in significant attenuation of DSS-induced weight loss, improved clinical parameters, and faster and more evident histological recovery compared to colitis controls. Specifically, DSS-induced colitis resulted in a marked pro-inflammatory response, with a significant increase in the expression of IL-1β, TNF-α, IL-6, MIP-2, and IFN-γ, along with a reduction in IL-10 levels. Treatment with EcN OMVs substantially reduced the expression of key pro-inflammatory cytokines, with significant differences for IL-1β, TNF-α, and IL-17, and partially restored IL-10 levels, both at the transcriptional and protein levels. OMV action also extended to markers of intestinal barrier function. Treatment restored trefoil factor (TFF)-3 expression to healthy levels and showed a tendency toward increased occludin, while ZO-1 was not significantly altered. OMVs also corrected the altered regulation of matrix metalloproteinases (MMPs) observed in colitic mice, significantly reducing MMP-9 expression and normalizing MMP-2. Concomitantly, a marked reduction in cyclo-oxygenase (COX)-2 and inducible nitric oxide synthase (iNOS) expression was observed, as confirmed at the protein level for the latter enzyme. Symptom-wise, pretreatment with OMVs attenuated the severity of DSS-induced colitis. Indeed, treated mice showed less weight loss, significantly lower DAI scores, and reduced intestinal edema. Macroscopic and histological analyses confirmed a clear protective effect, with less mucosal ulceration, preservation of goblet cells, reduced inflammatory infiltration, and significantly improved histological scores [155]. Moreover, Choi et al. explored the potential of *Lactobacillus paracasei*-derived extracellular vesicles (LpEVs) to modulate the inflammatory response, both in cell cultures and in a mouse model of colitis. The authors evaluated the effects of LpEVs on LPS-induced inflammation in HT29 human colorectal cancer cells and on DSS-induced colitis in C57BL/6 mice. In the mouse models, colitis was induced by administering 2% DSS, and disease severity was monitored using established clinical parameters: weight change, colon length, DAI, and survival rate. In vitro studies, LpEVs demonstrated a strong ability to modulate inflammation. Indeed, LPS-induced reductions in the expression of pro-inflammatory cytokines such as IL-1α, IL-1β, IL-2, and TNF-α occurred, while simultaneously increasing the production of protective cytokines such as IL-10 and TGF-β. HT29 cells also exhibited a decrease in the activation of key proteins involved in the inflammatory cascade, such as COX-2, iNOS, and NF-κB, as well as a reduction in NO levels, a further indicator of inflammatory stress. The in vivo results were fully consistent with the cellular findings. Oral administration of LpEV significantly attenuated the effects of DSS. Treated mice lost less weight, maintained greater colon length, and showed lower DAI scores compared to colitic controls. In summary, the anti-inflammatory effects of LpEVs in LPS-treated HT29 cells were completely abolished, shifting the response toward a pro-inflammatory phenotype. This data once again underline the ability of these vesicles to influence intestinal homeostasis [156]. Certain components present on the surface of EVs play a key role in modulating intestinal inflammation. Shen et al. demonstrated that *Bacteroides fragilis* incorporates capsular polysaccharide A (PSA) onto its OMVs, conferring strong immunomodulatory activity capable of preventing experimental colitis. DCs recognize PSA delivered by OMVs via the TLR2 receptor, activating a cascade that leads to the expansion of Treg cells and increased production of anti-inflammatory cytokines. This protective response requires the activity of the Gadd45α protein, involved in the response to cellular damage. Consistent with this, DCs treated with PSA-containing OMVs are effective in preventing experimental colitis. Conversely, DCs lacking Gadd45α are unable to promote the activation of Treg cells or to suppress the production of pro-inflammatory cytokines, rendering them incapable of limiting the progression of the disease [157].

Numerous studies have now highlighted the great potential of OMVs in modulating gastrointestinal inflammation. Bacterial EVs are emerging as an innovative therapeutic solution, capable of offering distinct advantages over more complex interventions such as FMT, BCT, or phage therapy. OMVs allow for a much more targeted approach: they can deliver specific molecules directly to the desired site, reducing the side effects often associated with therapies that act in a broader and less controlled manner. Unlike phage therapy, they do not pose risks related to bacterial resistance, and being non-living entities, they eliminate the concerns of pathogen transmission typical of FMT. Furthermore, their production is more standardized, overcoming the variability of therapies based on human donors [158]. Another key advantage of OMVs is their ability to directly interact with the immune system, promoting the restoration of microbial balance and improving intestinal health without relying on the colonization of new bacteria, as is the case with live probiotic consortia. There are also limitations: in conditions of very severe dysbiosis, OMVs may not be able to compete with a severely altered microbiota and therefore not achieve the full restorative effect that FMT can offer. Furthermore, their production requires advanced technologies and an even deeper understanding of the complex interactions with the immune system and long-term effects. Their potential use in personalized treatments also presents additional challenges [159]. Despite these obstacles, OMVs represent one of the most promising frontiers of microbiome medicine (Table 3).

### 4.4. Phage Therapy

Bacteriophages are viruses that specifically infect bacteria, playing a vital role in regulating bacterial populations and maintaining microbial balance. Bacteriophages represent highly specific tools for the targeted modulation of the human microbiota. Thanks to their high diversity and ability to selectively target pathogens without altering beneficial communities, phages enable personalized, low-toxicity interventions. Their self-replicating mode, ability to exploit both lytic and lysogenic cycles, and their potential role as vectors to introduce or silence bacterial functions highlight their value as innovative tools for regulating the microbiota and for the development of next-generation therapeutic approaches [160]. Several preclinical and clinical studies are exploring phage therapy for the treatment of diseases, particularly those related to dysbiosis. In particular, targeted phage therapy against AIEC strains, bacteria closely implicated in dysbiosis and the pathogenesis of CD, represents one of the most promising strategies for restoring intestinal microbial balance. EcoActive, an FDA-approved product, represents one of the most effective approaches. It is a cocktail composed of seven lytic phages selected to specifically target AIEC strains without compromising the commensal microbiota. Preclinical studies have demonstrated that this phage mix possesses high in vitro efficacy, proving active against 95% of the 210 AIEC clinical strains tested. Its specificity is significantly superior to that of conventional antibiotics, as it is unable to lyse the 43 strains belonging to the main bacterial genera typical of a healthy microbiota. This high specificity therefore reduces the risk of dysbiosis, one of the main limitations of traditional antimicrobial therapies. In vivo evaluation has further confirmed EcoActive’s good safety profile. Indeed, prolonged administration in healthy mice did not induce significant alterations in the bacterial community, as evidenced by metagenomic analyses. In mouse models colonized with the AIEC LF82 strain and subjected to induced colitis, phage therapy showed a dose- and time-dependent protective effect. Twice-daily treatment for 15 d resulted in a marked reduction in clinical and histological signs of inflammation. Overall, these findings support AIEC-targeted phage therapy as a safe, specific, and effective approach to reducing pathogenic bacterial load and modulating intestinal inflammation in patients with CD. Based on preclinical results, a Phase I/IIa clinical trial is currently underway to evaluate its safety and efficacy in patients [161]. Similarly, phage therapy targeting *Klebsiella pneumoniae* has emerged as a promising strategy for mitigating intestinal inflammation associated with IBD. Recent preclinical studies have demonstrated that a combinatorial phage approach can effectively suppress pathogenic *Klebsiella pneumoniae* strains, particularly the antibiotic-resistant Kp2 clade, present in IBD patients. Through an iterative isolation and validation process, researchers identified a consortium of five phages, MCoc5c, 8M-7, 1.2–3 s, KP2-5-1 and PKP-55, that consistently reduced bacterial burden in vitro and in murine models, including mucoadherent populations, without harboring virulence or antibiotic resistance genes (clinical-stage product). Optimal oral administration regimens further enhanced phage replication and bacterial clearance [162]. A recent preclinical study demonstrated that phage therapy targeting Kp2 strains of *Klebsiella pneumoniae* can ameliorate inflammation in IBD models. When administered orally, the combination of five phages, MCoc5c, 8M-7, 1.2–3 s, KP2-5-1, and PKP-55, significantly reduced Kp2 levels in both feces and mucosal-adherent bacteria in mouse models (clinical-stage product). Specifically, phage therapy provided sustained suppression of Kp2 in long-term models of subclinical inflammation without the emergence of phage-resistant mutants. This treatment also attenuated colonic inflammation, as evidenced by a reduction in IFN-γ^+^ CD4^+^ T cells and lower levels of pro-inflammatory cytokines and chemokines (IL-9, IL-15, IL-17). These results highlight the potential of targeted phage combinations to modulate and mitigate pathogen-induced intestinal autoinflammation, supporting their future translational application and complementing the ongoing Phase I clinical trials evaluating their safety in humans [163]. Nale et al. evaluated a cocktail composed of four temperate phages—phiCDHM1, phiCDHM2, phiCDHM5, and phiCDHM6—in bioreactors containing cultures of a clinical strain of *Clostridium difficile* ribotype 014/020, inoculated with combined fecal slurries from healthy volunteers. Prophylactic treatment resulted in an approximately 6-log reduction in *Clostridium difficile* abundance after just 5 h, while complete eradication was observed after 24 h in both the prophylactic and therapeutic regimens. Importantly, viability assays showed that administration of the cocktail did not compromise *Enterococci*, *Bifidobacteria*, *Lactobacilli*, total anaerobes, and *Enterobacteria*. However, in the vessel treated exclusively with phages, an approximately 2-log increase in *Enterobacteria*, *Lactobacilli*, and total anaerobes was observed compared to the other treatments, suggesting possible modulatory effects on the microbiota. Subsequent metagenomic analyses confirmed the selective impact of phages on the gut microbiota. These results support the therapeutic potential of optimized phage cocktails in the treatment of CDIs. Furthermore, the increase in specific commensals observed in the phage-only control suggests an additional protective effect, potentially useful in preventing recolonization by *Clostridium difficile* and stabilizing the gut microbial ecosystem [164]. The same research group analyzed the host ranges of six myoviruses and one siphovirus, identified as phiCDHM1, phiCDHM2, phiCDHM3, phiCDHM4, phiCDHM5, and phiCDHM6, on 80 strains representing 21 clinically relevant ribotypes, observing complementary coverage: single phages lysed 18 ribotypes and 62 total strains (clinical-stage product). Treatments with single phages against ribotypes 076, 014/020, and 027 initially reduced bacterial load but were followed by the emergence of resistant colonies. These colonies, however, remained susceptible to infection by unrelated phages. Conversely, specific phage combinations induced complete lysis of *Clostridium difficile* in vitro, preventing the emergence of resistant or lysogenic clones. In experimental hamster models, oral administration of optimized phage cocktails reduced intestinal colonization as early as 36 h post-infection, with recovery of free phages in the intestinal tract. In challenge models, treatment delayed the onset of symptoms by approximately 33 h compared to untreated animals. These data confirm the therapeutic potential of phage combinations, particularly phiCDHM1, phiCDHM2, phiCDHM3, phiCDHM4, phiCDHM5, and phiCDHM6, for the treatment of CDIs, highlighting both their ability to completely eradicate the pathogen and prevent the emergence of resistance [165]. Although numerous studies highlight the potential of phage therapy, some clinical trials have reported disappointing results. In particular, a clinical trial conducted on Bangladeshi children hospitalized for acute bacterial diarrhea evaluated the efficacy of a cocktail of T4-like coliphages (AB2, 4, 6, 11, 46, 50, 55; JS34, 37, 98, D1.4) administered orally for 4 d, compared to a placebo. The goal was to reduce stool frequency and the need for rehydration, as well as evaluate the safety and intestinal replication of the phages. The main results showed that the treatment was safe, with no adverse events attributable to the oral phages. The presence of coliphages in the stool was increased in treated children, but there was no evidence of significant intestinal replication. Only 60% of patients had diarrhea microbiologically confirmed by *Escherichia coli*, primarily enteroaggregative *E. coli* (ETEC), and the proportion of *Escherichia coli* in the fecal microbiota was less than 5%. Phage-susceptible *Escherichia coli* colonies were present in only half of the children. Fecal ETEC titers showed transient peaks, below the threshold required for effective T4 phage replication in vitro. Consequently, an interim analysis of 120 patients showed no clinical improvement in diarrhea parameters compared to standard therapy. Fecal microbiota analysis showed an increase in *Streptococcus* (primarily *Streptococcus gallolyticus* and *Streptococcus salivarius*), the abundance of which correlated with quantitative diarrhea outcomes, but genomic sequencing did not detect any virulence genes. This study suggests that, although oral administration of coliphages is safe, their therapeutic efficacy may be limited by insufficient phage coverage and excessively low levels of the target pathogen in the intestinal tract. The findings highlight the need to investigate further phage-bacterium interactions in vivo and the role of *Escherichia coli* in childhood diarrhea to make phage therapy clinically effective [166].

Phage therapy offers several advantages over traditional antibiotic approaches. Among the main advantages are target specificity, which minimizes off-target effects and preserves beneficial microbial communities, and the potential for synergism with other treatments, including antibiotics or probiotics, to enhance therapeutic efficacy. Furthermore, phages can self-replicate in the presence of the target bacteria, allowing for effective treatments at relatively low doses and reducing the risk of toxicity. However, the approach has some significant limitations. Phages can select resistant bacterial strains through various processes. Exposure to phages rapidly eliminates bacterial strains susceptible to infection, favoring the selection of resistant strains. The most common mechanism of phage resistance is related to the acquisition of mutations in the bacterium that alter or eliminate the surface receptors used by the phage for adsorption. Activation of phage defense systems (CRISPR-Cas systems, restriction-modification systems, etc.) leads to the death of the infected cell, preventing phage replication. Furthermore, changes in metabolic state or growth rate can reduce the replicative efficiency of the phage, making the bacterium less permissive to infection. In this context, the use of dynamic phage cocktails or phage alternation represents a key approach to limiting the selection of resistance and increasing the sustainability of phage therapy over time [167]. Phage selection must consider clinically relevant type coverage, in vivo replication capacity, and compatibility with the patient’s gut microbiota. Furthermore, the need to ensure precise targeting poses logistical and regulatory challenges, such as high target specificity, large-scale production, preparation standardization, and clinical validation. Recent advances in phage engineering and synthetic biology are opening new avenues to overcome some of these critical issues. Strategies such as modulating the phage’s spectrum of action, genome editing, and synthetic phage design enable more precise targeting of bacterial pathogens, reducing adverse effects. However, the need to ensure high specificity still poses significant logistical and regulatory challenges, particularly related to large-scale production, preparation standardization, and clinical validation, all of which are essential to ensure safety, efficacy, and reproducibility in the clinical setting [168]. In summary, while phage therapy represents a promising strategy for the treatment of bacterial infections, its effective clinical application still requires in-depth studies on optimized cocktails, in vivo interactions with the microbiota, and personalized protocols capable of addressing the complexity of bacterial resistance and pathogen variability [169] (Table 4).

## 5. Concluding Remarks and Future Perspectives

The gut microbiota is now recognized as a key determinant of human health, with a crucial role in the development and progression of metabolic, immune, inflammatory, neuropsychiatric and infectious diseases. Dysbiosis, induced by factors such as diet, lifestyle, psychosocial stress, environmental exposures and medication use, alters the diversity and functions of the microbial community, promoting systemic inflammation, immune imbalances and metabolic alterations. The interaction between microbial metabolites, host genetics and epigenetic regulation generates a dynamic feedback loop, emphasizing the bidirectional nature of microbiota-host interactions and their central role in disease susceptibility. Insight into these complex interactions demands integrated multi-omic approaches, combined with AI and high-resolution sequencing, to map microbial composition, functional pathways, and host responses in detail. These tools will enable the identification of robust microbial biomarkers to assess disease risk, clinical progression, and treatment response, paving the way for personalized, evidence-based interventions. Emerging strategies for adapting microbiota, such as FMT, BCT, phage therapy, and OMVs, provide promising tools for restoring eubiosis. These approaches have advantages and disadvantages. The main advantages include the ability to rapidly restore microbial diversity, modulate the immune system, and correct metabolic imbalances. On the other hand, limitations and disadvantages include variability in individual responses, unexpected adverse effects, complexity in standardizing preparations, and high costs. Furthermore, the lack of optimized protocols and long-term studies limits our understanding of the durability and safety of these interventions (Table 5). Future research should focus on optimizing these approaches, ensuring safety, efficacy, reproducibility, and personalization, particularly in patients with severe or chronic dysbiosis. Longitudinal studies and clinical trials will be crucial to assess the durability, systemic effects, and long-term consequences of such interventions. Multi-omic screening approaches, combined with advanced ML models, are emerging as key tools for identifying the most promising microbial strains, enabling the linking of precise microbial and metabolic signatures to patient clinical responses. This strategy fosters a greater understanding of the mechanisms governing the interaction between microbiota and health and enables the design of tailored probiotic and prebiotic administration. One of the most promising aspects is personalized microbiota therapy, based on the unique characteristics of each patient’s microbial profile. The integration of multi-omic data with AI allows for detailed mapping of the microbiota’s composition, function, and metabolic interactions, providing evidence for optimized treatments. This approach allows for therapeutic response prediction, minimizing side effects, and limiting the proliferation of unwanted strains. Furthermore, by combining clinical, metabolomic, and genomic data with advanced predictive algorithms, it is possible to stratify patients and identify those who will benefit the most, thus strengthening the application of truly individual-centered precision medicine. Embedding knowledge about microbiota into precision medicine has the potential to revolutionize prevention, management, and healthcare, establishing gut microbiota modulation as a key component of individualized, evidence-based therapeutic strategies.

## Figures and Tables

**Table 1:** Preclinical and clinical studies on FMT.

Condition	FMT Preparation	Study Model/Phase	Main Outcomes	Reference
FMT via oral capsules (fresh bacterial preparations)	Encapsulated donor-derived microbiota	Pilot clinical trials	Practical alternative to colonoscopy/infusion; improved patient compliance; maintained microbial transfer efficacy	[130,131]
Skin diseases and metabolic disorders	Donor-derived stool microbiota	Clinical trials ongoing	Early evidence of beneficial microbiota reshaping; studies in progress	[127]
Psychiatric and neurodevelopmental disorders	Donor-derived stool microbiota	Clinical trials ongoing	Investigated for modulation of gut-brain axis; potential improvement in behavioral and psychiatric outcomes	[128]
rCDI	Donor stool, RBX2660 (Rebyota), key bacteria such as *Bacteroides*	Phase III clinical trial	Higher success rate than placebo (70.6% vs. 57.5%) in preventing recurrence; FMT rapidly alters gut microbiota; generally safe	[129]
Active UC	50 mL enemas/wk from healthy donors	Randomized, placebo-controlled, parallel study	Clinical and endoscopic remission in 24% of FMT vs. 5% placebo; increase in microbial diversity; remission influenced by disease duration	[132]
Mild-moderate UC	Nasoduodenal tube administration of donor FMT	Randomized, double-blind, controlled study	Per-protocol remission: 41.2% FMT vs. 25% control; microbiota in responders resembled donor; safe, no serious FMT-related adverse events	[133]
UC	Oral encapsulated frozen stool (cFMT) daily for 12 wk after colonoscopy induction	Randomized study	Safe and well tolerated; 2/6 patients achieved clinical remission; sustained changes in microbiota; changes in MAIT cell cytokine production correlated with response	[134]
UC	Donor-derived microbiota, various administration routes	Meta-analysis of 13 randomized controlled studies, 580 patients	FMT significantly increased clinical remission (RR 1.73; 95% CI 1.41–2.12; *p* < 0.00001) and endoscopic remission (RR 1.74; 95% CI 1.24–2.44; *p* = 0.001); adverse events not significantly different from control (RR 1.00; 95% CI 0.86–1.15)	[137]
rCDI	Donor-derived microbiota, various administration routes	Meta-analysis of 15 studies, 777 patients	High cure rates for single FMT (81%) and overall FMT (92%, *p* = 0.0015); 12% serious adverse events, mostly hospitalization, IBD-related surgery, or IBD flare	[138]

**Table 2:** Preclinical and clinical studies on BCT.

Condition	BCT Preparation	Study Model/Phase	Main Outcomes	Reference
CDI (antibiotic-induced dysbiosis)	Synthetic BC (derived from healthy donor stool)	Preclinical/Clinical pilot studies	Restored microbiota balance; comparable efficacy to FMT	[136]
rCDI	Synthetic stool substitute composed of selected strains: *Bacteroides ovatus*, *Bifidobacterium lingua*, *Lactobacillus casei* from a single healthy donor	Pilot study, 2 adult patients non-responsive to ≥3 antibiotic cycles; infusion via colonoscopy	Restoration of normal gut microbiota within 2–3 d; symptom-free for 6 mo; stable colonization of therapeutic bacteria (>25% of sequences up to 6 mo)	[137]
rCDI	13 species isolated from healthy donors via culturomics (*Acidaminococcus intestini*, *Bacteroides fragilis*, *B. ovatus*, *B. uniformis*, *Bifidobacterium longum*, *Clostridium scindens*, *Lactobacillus casei*, *L. gasseri*, *L. rhamnosus*, *L. parabuchneri*, *Parabacteroides distasonis*, *Propionibacterium avidum*, *Ruminococcus gnavus*; 5 × 10^8^ cfu/mL)	Clinical evaluation with metagenomic analysis; stool DNA sequenced pre-infusion and at 7, 15, 30, 90 d post-infusion	Active microbial repopulation; increase in *Firmicutes* and *Verrucomicrobia*, decrease in *Bacteroidetes* or *Proteobacteria* depending on baseline; effective trigger for gut recolonization	[138]
rCDI	SER-109 (Vowst), oral BCT based on *Firmicutes* spores	ECOSPOR III & IV, double-blind, randomized, placebo-controlled trials; adult patients post-antibiotics	Significant reduction in rCDI risk (RR = 0.32, 95% CI 0.18–0.58 at 8 wk); *Firmicutes* spore engraftment associated with secondary bile acid formation; mild-to-moderate adverse events; improved quality of life; reduced hospitalizations and ER visits	[139]
rCDI	General BCT approaches	Review/Preclinical & clinical studies	Advantages over FMT: controlled, safe, reproducible; use of stable and standardized strains. Limitations: efficacy influenced by strain competitiveness, potential acquisition of antibiotic resistance, less effective in severe dysbiosis	[140,141,142]

**Table 3:** Key studies evaluating the therapeutic potential of bacterial extracellular vesicles (EVs/OMVs) in intestinal inflammation.

Target Pathogen/ Condition	OMV/EV Preparation	Study Model/Phase	Main Outcomes	Reference
*Bacteroides fragilis* (ETBF vs. NTBF); intestinal homeostasis vs. pathogenicity	OMVs isolated from ETBF and NTBF; proteomic (HPLC-MS/MS) and metabolomic profiling (HPLC-MS/MS, GC-MS)	In vitro biochemical characterization; comparative proteomics, metabolomics, fluxomics	ETBF OMVs are enriched in cytoplasmic and stress-response proteins, showing pathogenic metabolic activity; NTBF OMVs are enriched in essential amino acids and glycolytic intermediates, supporting homeostasis. OMVs contain unique metabolites that are not present in cells. Mechanistic basis for dual beneficial versus pathogenic roles.	[144]
*Akkermansia muciniphila*; gut homeostasis, colitis, cancer immunotherapy	Purified OMVs from *A. muciniphila*	In vitro (epithelial uptake, immune activation) and in vivo (colitis model; colorectal cancer anti-PD-1 therapy)	OMVs restored dysbiotic microbiota, enhanced B-cell and DC activation, increased IgA, strengthened barrier (tight junctions and mucus), alleviated colitis, and improved anti-PD-1 immunotherapy efficacy.	[145]
*Lactobacillus kefirgranum* PRCC-1301; DSS colitis	EVs isolated from PRCC-1301	In vitro (Caco-2) and in vivo (acute DSS colitis; chronic colitis in IL-10 gene knockout in mice)	EVs reduced pro-inflammatory cytokines, restored tight-junction proteins (ZO-1, claudin-1, occludin), improved clinical and histological outcomes, and inhibited NF-κB signaling (decreased p-p65 and p-IκBα).	[146]
*Clostridium butyricum*; DSS colitis	Purified bacterial EVs	In vivo DSS-induced colitis; transcriptomics (RNA-seq) and microbiota sequencing	EVs modulated inflammatory and immune pathways (IL-17, TNF-α, MAPK, PI3K-Akt, NF-κB), altered microbiota composition (increased *Ruminiclostridium*, *Anaerofustis*, *Helicobacter*, *Alistipes*), improved barrier integrity, and more effectively suppressed inflammatory gene upregulation than whole bacteria.	[147]
*Escherichia coli* Nissle 1917; DSS colitis	Purified OMVs (5 µg/d orally)	In vivo DSS-induced colitis; pretreatment + recovery model	OMVs reduced weight loss, DAI, inflammatory cytokines (IL-1β, TNF-α, IL-6, IL-17), restored IL-10, normalized TFF-3, corrected MMP-2/MMP-9 imbalance, reduced COX-2 and iNOS, and improved histology (increased goblet cells, decreased ulceration, decreased infiltration).	[148]
*Lactobacillus paracasei* LPS-induced inflammation and DSS colitis	Purified EVs (LpEVs)	In vitro (HT29 + LPS) and in vivo DSS colitis in C57BL/6 mice	LpEVs reduced pro-inflammatory (IL-1α, IL-1β, IL-2, TNF-α) and increased protective cytokines (IL-10, TGF-β)	[149]

**Table 4:** Preclinical and Clinical Studies on Bacteriophage Therapy Targeting Gut-Associated Diseases.

Target/Condition	Study Model	Phage/Cocktail	Main Outcomes	Reference
AIEC in CD	Preclinical (in vitro and murine models)	EcoActive (7 lytic phages)	Effective against 95% of the 210 AIEC strains; high specificity, does not alter commensal microbiota; dose- and time-dependent reduction in intestinal inflammation in colitis models	[154]
*Klebsiella pneumoniae* (Kp2) in IBD	Preclinical (in vitro and murine models)	Combination of 5 phages: MCoc5c, 8M-7, 1.2–3 s, KP2-5-1, PKP-55	Reduction in fecal and mucosal bacterial load; sustained suppression of Kp2; reduction in IFN-γ^+^ CD4^+^ T cells and pro-inflammatory cytokines	[156]
*Clostridium difficile* (ribotype 014/020)	Preclinical (bioreactors, murine, hamster)	Cocktail 4 tempered phages: phiCDHM1, phiCDHM2, phiCDHM5, phiCDHM6	~6-log reduction in 5 h, complete eradication in 24 h; does not compromise commensals; modulates microbiota; prevents resistance emergence	[157]
*Clostridium difficile* (various ribotypes)	Preclinical (in vitro and hamster)	6 myovirus + 1 siphovirus: phiCDHM1-6	Complete lysis with combinations; prevention of emergency resistance; reduction in intestinal colonization and delay in the onset of symptoms by ~33 h	[158]
Children with acute bacterial diarrhea (Bangladesh)	Clinical, Phase I/II	Cocktail T4-like coliphages (AB2, 4, 6, 11, 46, 50, 55; JS34, 37, 98, D1.4)	Safe, no adverse events; increased coliphage in stool; no significant intestinal replication; no clinical improvement on diarrhea parameters	[159]

**Table 5:** Advantages and disadvantages/limitations of FMT, BCT, phage therapy and OMVs approaches.

Approach	Advantages	Disadvantages/Limitations
FMT	Rapid restoration of a eubiotic microbial community High efficacy in rCDI Immunomodulatory potential and possibility of remission in refractory patients Availability of less invasive oral capsules	Need for rigorous donor selection and characterization Risk of undetected pathogen transfer Variable clinical response influenced by donor and recipient profiles Uncertainties regarding long-term safety and risks for the immunocompromised
BCT	Standardized, controlled, and reproducible alternative to FMT Use of stable bacterial strains, avoiding heterogeneous fecal material Exclusion of viruses and unwanted pathogens (improved safety profile)	Response may be influenced by the competitive ability of the introduced strains. Less effective in cases of severe dysbiosis. Risk of acquiring antibiotic-resistant strains.
Phage Therapy	Specificity is only toward target pathogens without harming beneficial bacteria. Self-replicating ability	Synthesis of personalized cocktails for each patient Clinical data is still limited
OMVs	Targeted microbiota modulation and immune support without the use of live bacteria Ability to interact with the immune system and preserve the intestinal barrier Promote the growth of beneficial bacteria while suppressing pathogens	Complex and difficult manufacturing process Long-term effects not yet fully understood Current clinical data still limited compared to other approaches

## Data Availability

No new data were created or analyzed in this study. Data sharing is not applicable to this article.
